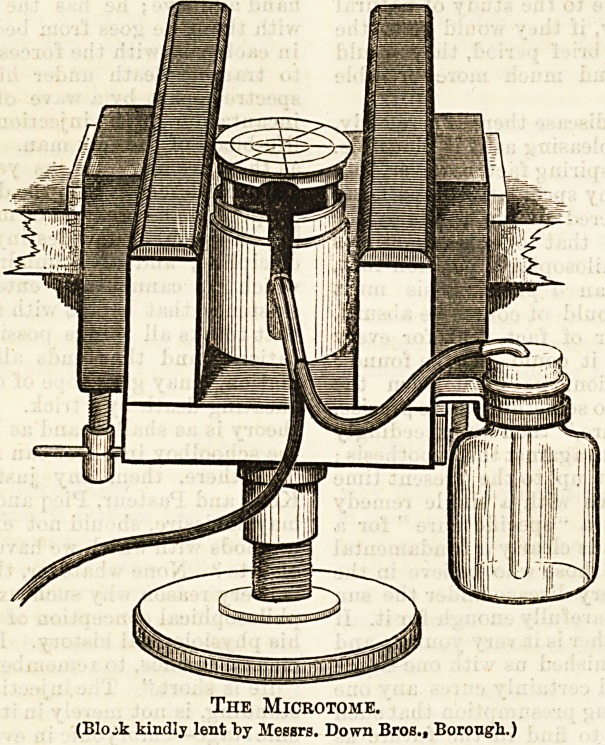# The Microscope in Medicine

**Published:** 1891-02-07

**Authors:** Frank J. Wethered


### The Microscope in Medicine.

By Frank J. Wethered, M.D.

### IV.—"CUTTING SECTIONS."

The specimens having been duly hardened as described in our last article, are now ready for embedding, preparatory to cutting.

There are two chief modes of embedding :—

I. In gum, combined with freezing.

II. In celloidin or paraffin. The former is the simplest and most speedy, and the one generally adopted, whilst the latter is more suitable for objects difficult to cut, such as the central nervous system, the eye, &c. Frazer's Modification of Cathcart's Microtome, described above, is suitable for either method.

We will first consider the manner of preparing and cutting frozen sections. The specimens which have been hardened by one of the processes detailed in the previous article, are removed from the alcohol or Müller's fluid, and soaked in water for several hours until all the hardening fluid has been removed. To do this effectually, the water should be frequently changed, and we may know that it has done its work in the case of the alcohol when the specimen sinks to the bottom of the containing vessel, and in the case of Müller's fluid when the washings are no longer coloured. When this stage is reached, the preparations are placed for twenty-four hours in a solution of gum, prepared according to the following formula : Colourless gum Arabic, two parts ; cold water, three parts. This must be well stirred until the gum is dissolved, and then a little thymol or carbolic acid added, as a preservative. One of the specimens is then removed to the plate of the microtome ; this is best managed by means of forceps, and a little of the gum solution should be poured over it. The ether spray apparatus is then set to work, but not too rapidly. Very soon a white ring will be observed to form at the edge of the gum, which gradually spreads until the whole is a frozen mass firmly fixed to the plate. The left hand then manipulates the micrometer screw. This must be first lowered until the top of the specimen is on a level with the glass guides of the microtome. The large plane-like knife is held firmly with the right hand, and passed quickly and evenly along the guides, the micrometer screw being raised a little at the end of each cut. When a large enough surface of the object has been exposed, the spray apparatus should again be worked, in order to assure that the frozen mass is firmly adherent to the plate.

The Microtome.

(Block kindly lent by Messrs. Down Bros., Borough.)

Sections are now cut by rapidly passing the knife along the guides, the thickness being regulated by the screw. When four or five sections have been collected on the knife they should be removed by carefully sweeping them off into a dish of water, either by means of a camel's hair brush or the finger, the knife being immediately dried with a soft cloth. Advice is sometimes given to moisten the knife, but this is quite sufficiently done by the thawing of the specimen. From time to time more ether must be sprayed underneath the plate, otherwise the gum will become loosened. The knife should not be driven perfectly squarely over the specimen, but slightly cross-wise, so as to expose a greater portion of its edge for cutting purposes. After use the knife should be again dried and passed a few times over a sharpening stone, especial care being used to remove all notches and secure a straight, sharp, and smooth edge.

Much better sections are obtained by embedding the specimens in celloidin or paraffin than by freezing. The choice between celloidin and paraffin as embedding media, is a difficult one. In the opinion of the writer, celloidin is to be preferred for ordinary work; it is easy to manipulate, and is suitable for all material, except that intended to show fatty changes.

Paraffin is advantageous for "series-cutting," and in preparing sections with the Cambridge Rocking Microtome it is absolutely essential. It is, however, not so easy to work with as celloidin, owing to the care with which it has to be prepared. In these papers, therefore, a description only will be given of the celloidin method.

Celloidin is usually sold in small shavings. A few of these are placed in a wide-mouthed bottle and a mixture of equal parts of absolute alcohol and ether is added, the mixture being well stirred until the consistency of thin mucilage is attained. The specimens which have been hardened in Müller's Solution must be thoroughly washed in water until the liquid is no longer coloured ; those which have been hardened in alcohol need not be so treated. They are then placed for twelve hours in a solution of equal parts of absolute alcohol and ether, after which they are transferred to the celloidin solution. Here also they must remain at least twelve hours. Small pieces of cork are then prepared, the size of the pieces naturally depending upon the size of the specimens. A very good plan is to cut an ordinary bung into six pieces, one surface being made perfectly smooth by means of a sharp knife.

After being duly saturated in celloidin, the specimens are removed one by one with forceps, and each placed upon one of the pieces of cork. They are allowed to remain for a few minutes until the material begins to set, when they are thrown into a large jar containing 80 per cent. alcohol. The weight of the specimens will be sufficient to keep them slightly submerged without the necessity of attaching weights to the corks. In twelve hours the sections may be cut, but the specimens may remain in the alcohol for any length of time.

The plate of Cathcart's Microtome is removed, and the clamp fixed in its place. Into this the cork is fixed and the micrometer screw lowered until the top of the specimen is on a level with the guides.

Sections are then cut in the same manner as was described with the frozen objects, but with this exception, the knife and celloidin must be kept almost swimming in dilute alcohol (60 per cent.); unless this is done the sections are very apt to curl up and break. After being cut they are transferred to a shallow dish containing water, when by reason of the rapid separation of the alcohol they immediately spread out and are then again removed to dilute spirit. Should any difficulty be found in making the sections lie flatly open, they should be placed for a few minutes in strong spirit, and then again transferred to the water. This plan generally succeeds admirably It is not applicable, however, to brittle sections; the only way to manage them is to carefully unfold them by means of a camel's hair brush whilst still on the knife, and then to float them off into alcohol. Occasionally specimens break so easily that it is extremely difficult to obtain sections. This may sometimes be accomplished by painting the specimen over with collodion, and then passing the knife over it very slowly and gently. For those who intend to do much of this kind of work, one of the foreign microtomes such as Reichert's will be found a great luxury.

In the next article the various methods of staining sections will be considered.

## Figures and Tables

**Figure f1:**